# Satisfaction of antiretroviral therapy services and its associated factors among adult clients attending antiretroviral therapy in Woliso town, Ethiopia

**DOI:** 10.1186/s12981-024-00593-9

**Published:** 2024-01-22

**Authors:** Bayisa Abdissa, Rahel Abdissa, Jiregna Derega, Senahara Korsa Wake

**Affiliations:** 1 Department of Public Health, College of Medicine and Health Science, Ambo University, Ambo, Ethiopia; 2 Department of Statistics, College of Natural and Computational Sciences, Ambo University, Ambo, Ethiopia

**Keywords:** Antiretroviral therapy, Client’s satisfaction, Factors

## Abstract

**Background:**

Antiretroviral therapy client satisfaction is a significant tool that enables to strengthen the quality of life of the clients. The study aimed to assess the satisfaction of clients with antiretroviral therapy services and its associated factors among adult clients attending antiretroviral therapy.

**Methods:**

A cross-sectional study was undertaken from 28 August to 27 October 2022. Data were analyzed by using Logistic regression.

**Result:**

Clients who did not attend formal education and attended health education information were significantly associated with satisfaction on antiretroviral therapy services.

**Conclusion:**

HIV care services should introduce systematic health education programs to improve satisfaction with antiretroviral therapy services.

## Introduction

The World Health Organization (WHO) defines antiretroviral therapy (ART) as the combination of three or more medicines used to treat human immune deficiency virus (HIV) infection [1]. ART will impact mortality, reduce fatalistic attitudes such as stigma and discrimination, promote increased voluntary HIV testing, and provide a rationale for making healthy living choices [2].

ART client satisfaction is a very significant tool that enables us to strengthen the quality of life of the clients and institutions who provide services to assess specific problems and challenges of clients which need to be addressed [3]. A satisfied ART patient is more likely to have a more long-lasting relationship with their physician, which improves compliance and ensures continuity of care [4]. The measurement of ART client satisfaction might support health managers in evaluating the performance of healthcare delivery systems and interventions [5]. Even though various measures have been taken to ensure ART client satisfaction, it is difficult to achieve universal.

This study aimed to assess client satisfaction with antiretroviral services and associated factors among adult clients attending an antiretroviral therapy clinic in Ethiopia Woliso town.

## Methods

### Study period, design, and population

The study was conducted in Woliso town which is located in Oromia region, Ethiopia. The study was conducted from August 28 to October 27, 2022. An institutional-based cross-sectional study design was used. All adult clients who were on ART and aged > 18 during the study period were eligible for this study.

### Data process and analysis

To get the overall client satisfaction, clients who were satisfied with greater than or equal to 75% were categorized as “satisfied,” and those who were satisfied below 75% of the items were categorized as “dissatisfied” [6]. Descriptive statistics were computed for the study variables and frequency distribution tables were used to describe the data. The impacts of independent variables (socio-demographic characteristics clinical, health facility, and health care provider-related factors) on client satisfaction with ART were examined using Logistic regression.

## Results

### Socio-demographic and clinical characteristics of the study participants

A total of 361 adult ART clients were included in the current study. The participants’ mean (SD) age was 42 (10) years in which more than half of them, 213 (59%), were older than 40 years. About 221 (61.2%) of the respondents were females and married. The mean (SD) duration in which HIV/AIDS clients received ART was 54 (55) months. Related to the differentiated service delivery model (DSDM), about 234 (64.8%) participants were on the appointment-spacing model (ASM).

### Level of adult client’s satisfaction with ART services

The overall client satisfaction with antiretroviral service was 54.6% (95% CI: 49.6–59.6). About 116 (32.1%) of the clients showed low satisfaction with the availability of educational material, 132 (36.6%) with visible direction, 190 (52.6%) the cleanness of latrines at the health centers and 197 (60.1%) with a clear explanation of prescribed drugs. Detailed client satisfaction measurements are displayed in Table 1.

**Table 1:** Level of clients' satisfaction with ART service at Woliso town health facilities

Variable	Level of client satisfaction n (%)				
	Very dissatisfied	Dissatisfied	Neutral	Satisfied	Very satisfied
Healthcare workers communication					
The way the provider welcomed me	10 (2.8)	45 (12.5)	9 (2.5)	184 (51.0)	113 (31.3)
The provider approach when they were examining	15 (4.2)	42 (11.6)	7 (1.9)	181 (50.1)	116 (32.1)
The way providers are talking to me	13 (3.6)	44 (12.2)	9 (2.5)	179 (49.6)	116 (32.1)
How the provider listened to my concerns	14 (3.9)	39 (10.8)	13 (3.6)	176 (48.8)	119 (33.0)
How provider respect my privacy	13 (3.6)	42 (11.6)	16 (4.4)	167 (46.3)	123 (34.1)
Client Confidentiality is observed in the ART	11 (3.0)	46 (12.7)	9 (2.5)	159 (44.0)	136 (37.7)
Health center physical environment					
Cleanness of health center	18 (5.0)	49 (13.6)	17 (4.7)	144 (39.9)	133 (36.8)
Cleanness of latrine	73 (20.2)	76 (21.1)	22 (6.1)	108 (29.9)	82 (22.7)
Visible directions for clients	138 (38.2)	71 (19.7)	20 (5.5)	62 (17.2)	70 (19.4)
Availability of educational materials	174 (48.2)	57 (15.8)	14 (3.9)	52 (14.4)	64 (17.7)
Sufficiency of service-giving rooms	46 (12.7)	67 (18.6)	23 (6.4)	122 (33.8)	103 (28.5)
Diagnosis and treatment factors					
laboratory test available during the visit	12(3.3)	51(14.1)	15(4.2)	110(30.6)	173(47.9)
Obtain drugs at pharmacies	17(4.7)	46(12.7)	12(3.3)	112(31.0)	174(48.2)
The clear explanation given for drugs	93(25.8)	42(11.6)	9(2.5)	84(23.3)	133(36.8)

### Results of logistic regression

The results of logistic regression illustrated that educational status, DSDM, waiting time, and health education information are significantly associated with adult ART client satisfaction. Clients who had not attended formal education were 86.2% times (AOR = 0.138, 95% CI: 0.062-0.310) less likely to be satisfied with ART services compared to clients who had college and above. Clients who attended health education information were 3.29 times more likely to be satisfied (AOR = 3.291, 95% CI: 1.91-5.66) than clients who did not attend health education information (Table 2).

**Table 2:** Associated factors of adults client satisfaction with ART service at Woliso town health facilities, Ethiopia

Variable	Variable category	COR (95% CI)	AOR (95% CI)	P. value
Educational status	No formal education	0.16 (0.087–0.324)*	0.138 (0.062–0.310)*	0.001*
	Primary	0.872 (0.487–1.56)	0.489 (0.239–1.001)	0.05
	Secondary education	1.003 (0.547–1.83)	0.580 (0.275–1.222)	0.152
	College and above	1	1	1
Health education information	Yes	4.300 (2.736–6.757)	3.291 (1.912–5.663)*	0.002*
	No	1	1	1
Waiting time	< 30 min	7.050 (4.358,11.404)	2.692 (1.421,5.102)*	0.002*
	> = 30 min	1	1	1
Differentiated service delivery model (DSDM)	ASM	8.602 (5.203–14.221)*	4.327 (2.210–8.475)*	0.001*
	3MMD	1	1	1

*COR* crude odds ratio, *AOR* adjusted odds ratio

^*^Statistical significance (*P* ≤ 0.05), 1.00 = reference

## Discussion

This study revealed that 54.6% [95% CI 49.6–59.6] of adult clients were satisfied with ART services given at Woliso Town. This is consistent with previous studies in Mizan-Tepi and West Wolega Zone [7, 8]. However, this finding was lower than the study reported from Gonder Town (75.4%), and Jimma town (89.6%) [9, 10].

The statistically significant associations were found between overall client satisfaction and respondents’ education level, differentiated service delivery model, waiting time, and health education information. Clients who had not attended formal education were 86.2 percent times less likely to be satisfied compared to those who had attended formal education. This is consistent with the studies conducted in Nigeria, Mizan Tepi, and Gondar University referral hospitals in Northwest Ethiopia [4, 7, 11]. On the other hand, clients who had attended health education information had higher odds of being satisfied; they were 3.2 times more likely to be satisfied than those who had not attended health education information. This is consistent with the studies conducted in Nigeria, Eastern Ethiopia, and Addis Ababa Zewditu Hospital [4, 12, 13].

In terms of waiting time, clients who waited less than 30 min to get services were 2.6 times more likely to be satisfied than those who waited more than or equal to 30 min. This is congruent with the studies conducted in Anambra State, Nigeria; Diredawa, eastern Ethiopia, and Hadiya Zone southern Ethiopia [14–16].

Related to the DSDM, clients who were on the ASM were 4.33 times more likely to be satisfied than clients who were on 3MMD during their last follow-up visits. Regarding the adherence status of clients, there was no association between overall client satisfaction and adherence status among adult clients attending ART clinics in Woliso town. In contrast, a study conducted in Africa found that clients who had good adherence were moderately satisfied compared to those who had poor adherence [17].

## Conclusion

There was a moderate level of overall client satisfaction with the antiretroviral services offered in the hospital. HIV care services should introduce systematic health education programs to improve satisfaction with antiretroviral therapy services.

## Data Availability

The datasets used during the current study are available from the corresponding author at a reasonable request via bayoabdi@gmail.com.
